# Flood inundation assessment for the Hanoi Central Area, Vietnam under historical and extreme rainfall conditions

**DOI:** 10.1038/s41598-018-30024-5

**Published:** 2018-08-22

**Authors:** Pingping Luo, Dengrui Mu, Han Xue, Thanh Ngo-Duc, Kha Dang-Dinh, Kaoru Takara, Daniel Nover, Geoffrey Schladow

**Affiliations:** 1 Key Laboratory of Subsurface Hydrology and Ecological Effects in Arid Region, Ministry of Education, Chang’an University, Xi’an, China; 2 School of Environmental Science and Engineering, Chang’an University, Xi’an, China; 3 Institute for Studies of the Global Environment, Sophia University, Tokyo, Japan; 4 Department of Space and Aeronautics, University of Science and Technology of Hanoi (USTH) Vietnam, Academy of Science and Technology (VAST), Hanoi, Vietnam; 5 Faculty of Hydrology, Meteorology and Oceanography, VNU University of Science, Hanoi, Vietnam; 6 Disaster Prevention Research Institute (DPRI), Kyoto University, Uji, Kyoto Japan; 7 School of Engineering, University of California – Merced, 5200 Lake R, Merced, CA USA; 8 Department Civil and Environmental Engineering, UC Davis, Ghausi Hall, One Shields Ave, Davis, CA USA

## Abstract

Flash floods have long been common in Asian cities, with recent increases in urbanization and extreme rainfall driving increasingly severe and frequent events. Floods in urban areas cause significant damage to infrastructure, communities and the environment. Numerical modelling of flood inundation offers detailed information necessary for managing flood risk in such contexts. This study presents a calibrated flood inundation model using referenced photos, an assessment of the influence of four extreme rainfall events on water depth and inundation area in the Hanoi central area. Four types of historical and extreme rainfall were input into the inundation model. The modeled results for a 2008 flood event with 9 referenced stations resulted in an R^2^ of 0.6 compared to observations. The water depth at the different locations was simulated under the four extreme rainfall types. The flood inundation under the Probable Maximum Precipitation presents the highest risk in terms of water depth and inundation area. These results provide insights into managing flood risk, designing flood prevention measures, and appropriately locating pump stations.

## Introduction

Climate change has likely increased the frequency of heavy precipitation over the past two decades over many areas of the globe^1^. Extreme rainfall events have led to dramatic damage to human life and property, most seriously by contributing to urban flooding^2,3^. High population densities and low-lying topography in urban parts of Southeast Asia make them uniquely vulnerable to extreme rainfall and consequent floods. Notable examples of recent flooding in Southeast Asia include Hanoi in 2008^4^, Bangkok in 2011^5^, Beijing in July 2012^6^, and many large southern cities in China in July 2017^7^. The increasing risk of flood disasters makes analysis of water depth and inundation area all the more urgent as cities increasingly demand flood management strategies.

Many modeling tools and techniques have been developed to analyze flood inundation and address this knowledge gap. Spatially, floods have been analyzed at many scales. At the subnational scale, analyses of floods have been conducted for urban parts of Bordeaux, France^8^, the 1994 flood of Alba, Piedmont, Italy^9^, metropolitan areas of Charlotte, North Carolina, USA^10^, Guangzhou, China^11^ and the Dutch polder area^12^. At regional and national scales, analyses include a review of trend analysis and climate change impacts on floods in Europe^13^, incorporating the effect of climate change on flooding in Bangladesh^14^, flood dynamics in urbanized landscapes of north-eastern Italy^15^, and national scale flood hazard mapping^16^. Global-scale river flood vulnerability analyses have also been conducted^17^. Rainfall-runoff floods were also analyzed at the basin scale from 2.6 to 26000 km^2^ in the United States and Puerto Rico^18^. Flood events have been studied at the basin scale^19–21^ and in urban areas^5,22,23^. Flood simulation methods include flood frequency analysis^10,24–27^, process-based flood models^28–31^and satellite remote sensing^32–35^. Rainfall-runoff-inundation models were used to simulate the 2011 Thailand floods at the river basin scale^36^. Hydrological modelling was used for large-scale flood inundation forecasting linked to the semi-distributed Variable Infiltration Capacity (VIC) hydrologic model and LISFLOOD-FP hydrodynamic model^31^. It is essential to study urban floods under extreme rainfall using 2-dimensional flood inundation models to develop detail data about urban conditions and provide scientific support for urban flood management.

The Flo-2D model has been used for simulating flood events in many contexts. Global Climate Models (GCMs) have been used as input data to the Flo-2D model to predict future flood events^37^. Low impact development effects on flood inundation of urban areas have been analyzed to develop rainfall harvesting systems and permeable pavements which have a significant impact on mitigating flood inundation risk^38^. Flood risk assessment of informal urban settlements has also been conducted using assessment of structure fragility and the Flo-2D model^39^. Remotely sensed images have also been used to achieve spatial parameterization of Manning’s roughness coefficient for the Flo-2D model^40^. Uncertainty of flood mapping assessments with Flo-2D has been evaluated using HEC-RAS and LISFLOOD-FP based on benchmark analyses and assessment of real-world cases and used to calculate additional uncertainties due to data scarcity^41^. The first-order perturbation method based on a Karhunen-Loevè expansion was tested to analyze uncertainty of Flo-2D analyses with a random floodplain roughness field^42^.

The objectives of this study are to set up a flood inundation model (Flo-2D model), analyze water depth and inundation area under four historical and extreme rainfall types, and identify sustainable approaches to urban flood management under present conditions and under climate change conditions. Referenced photos were used to calibrate flood simulation results for the Hanoi central area in 2008. The  four historical and extreme rainfall types include the historical rainfall of 2008, the historical maximum two-day rainfall, probable precipitation within return period of 200 years and probable maximum precipitation. Detail information about water depth and inundation area under extreme precipitation reveals areas prone to urban floods and provides scientific guidance for designing and implementing flood prevention structures and developing future flood prevention plans.

## Results

### Study area and Data

**Figure 1:**
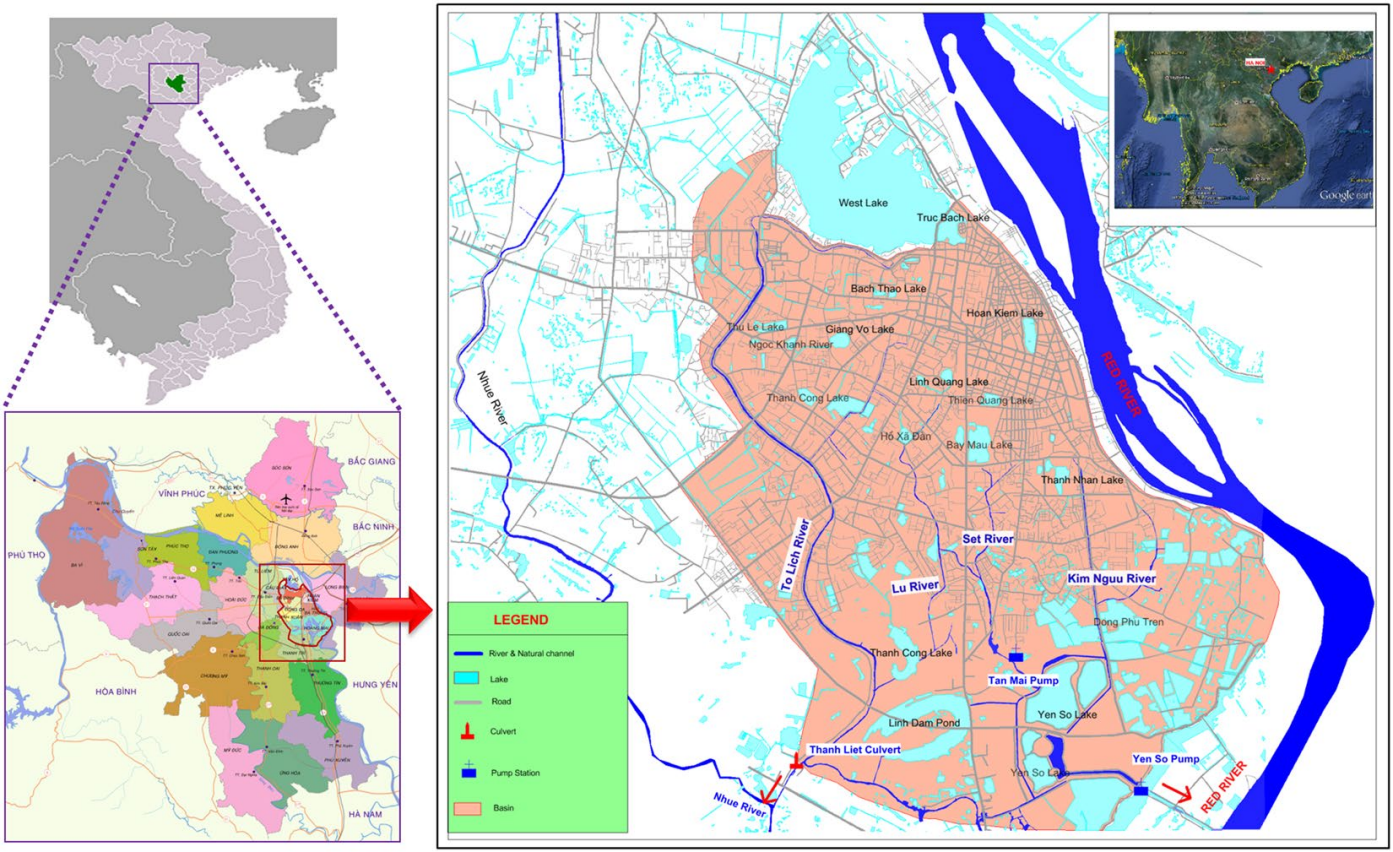
Location map of Hanoi central area for the flood inundation simulation (Source: Southeast map is obtained from Google Earth, Administrative divisions of Hanoi is obtained from Wikipedia, https://en.wikipedia.org/wiki/Hanoi, The RAW data of central area map is from project of “Collection and Analysis of Data Related to Flood and Inundation in Hanoi Capital” Kha Dang-Dinh re-edited this map.).

Hanoi is the capital of Vietnam and has seen increasing urbanization and economic development during recent decades. Hanoi is located Northwest of the Red River delta from 20°53′ to 21°23′ north latitude, and from 105°44′ to 106°02′ east latitude, adjacent to Thai Nguyen, and Vinh Phuc to the North, Ha Nam to the South, Phu Tho and Hoa Binh to the West, and Bac Giang, Bac Ninh, and Hung Yen to the East. After the administrative boundary expansion in August 2008, Hanoi has an area of 3,344.70 km^2^ with a total population of approximately 7.74 million. The Hanoi central area is located at the right side of the Red River (Fig. 1). The city is now urbanizing quickly, with a continuous flow of immigrants from peri-urban and rural areas. The Hanoi Central Area is approximately 76.26 km^2^ with an average annual temperature of 24 °C at an elevation of 5 to 20 m and an average annual total rainfall of 1676 mm. Population growth and economic development have put strong pressure on the environment and natural resources and the city’s infrastructure as well. In fact, the pace of urban infrastructure development has lagged population growth and economic development. A major emerging issue regarding insufficiency of city infrastructure is the lack of safe drinking water supply and sewerage coverage in newly urbanized areas, which has led to serious environmental pollution in the receiving water bodies of the city.

Between October 30 and November 1, 2008, a flood triggered by torrential rains caused a flood disaster which led to significant human casualties and economic loss^4,43^. This flood affected north and central Vietnam, as well as southern parts of China and caused the death of 92 people. Up to one meter of water flooded the city’s streets, and urban transportation was halted. Food prices, especially those of meat and vegetables, reached exorbitant levels in the city as the rains destroyed crops and livestock and crippled transportation corridors. The total economic loss due to this flood exceeded 177 million USD.

The four main rivers in this catchment (To Lich, Lu, Set and Kim Nguu) flow from West to East, discharging into the Nhue River through the Thanh Liet Culvert^44^. Flow in the system is mainly generated from local rainfall with strong seasonal variation. The To Lich River is largely responsible for draining discharge from the city during heavy rain^45^. The Yen So pumping station is the primary station for draining water from the central area whenever heavy rain occurs. The pumping station was built in 1998, including six sluice gates of 3 m width and a maximum water level of 11 m. The designed pumping capacity in 2008 was 45 m^3^/s, and it has been expanded to 90 m^3^/s after the second phase of construction, which was finished in September 2010. The pumping capacity of 45 m^3^/s was used in the Flo-2D model (described below) as the flood events took place in 2008.

**Figure 2:**
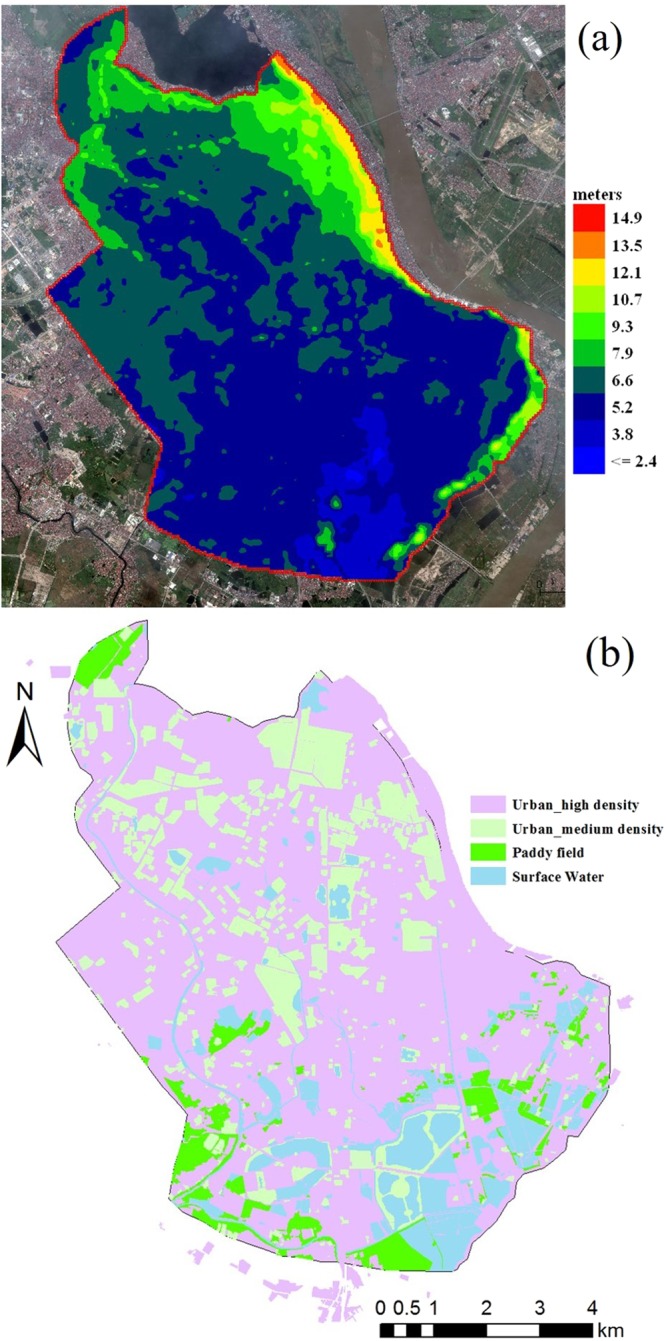
DEM map (**a**) (made by Flo-2D Pro, www.flo-2d.com) and Land use map (**b**) (made by ArcMap 10.2, www.esri.com)of Hanoi central area (Source: DEM data is modified from the 30 meter DEM data of USGS, background image map is obtained from the open source of Google Earth which data is from SIO, NOAA, U.S. Navy, NGA, GEBCO and image copyright belongs to 2012 Google. Land use map is drawn by Han Xue, the original land use data is provided by Kha Dang-Dinh).

Approximately 30-meter mesh Digital Elevation Model (DEM) data (Fig. 2a) was collected from the original data of the Shuttle Radar Topography Mission (SRTM) 1 Arc-Second Global^46^. Land use data from 2010 (Fig. 2b) was collected from the Hanoi Urban Planning Institute. Historical daily rainfall data in Hanoi for 1961–2008 was recorded by the National Hydro-Meteorological Services of Vietnam. In the flood inundation model, we only consider the river channel based on elevation due to a lack of cross-section data.

### Flood inundation simulation results

**Figure 3:**
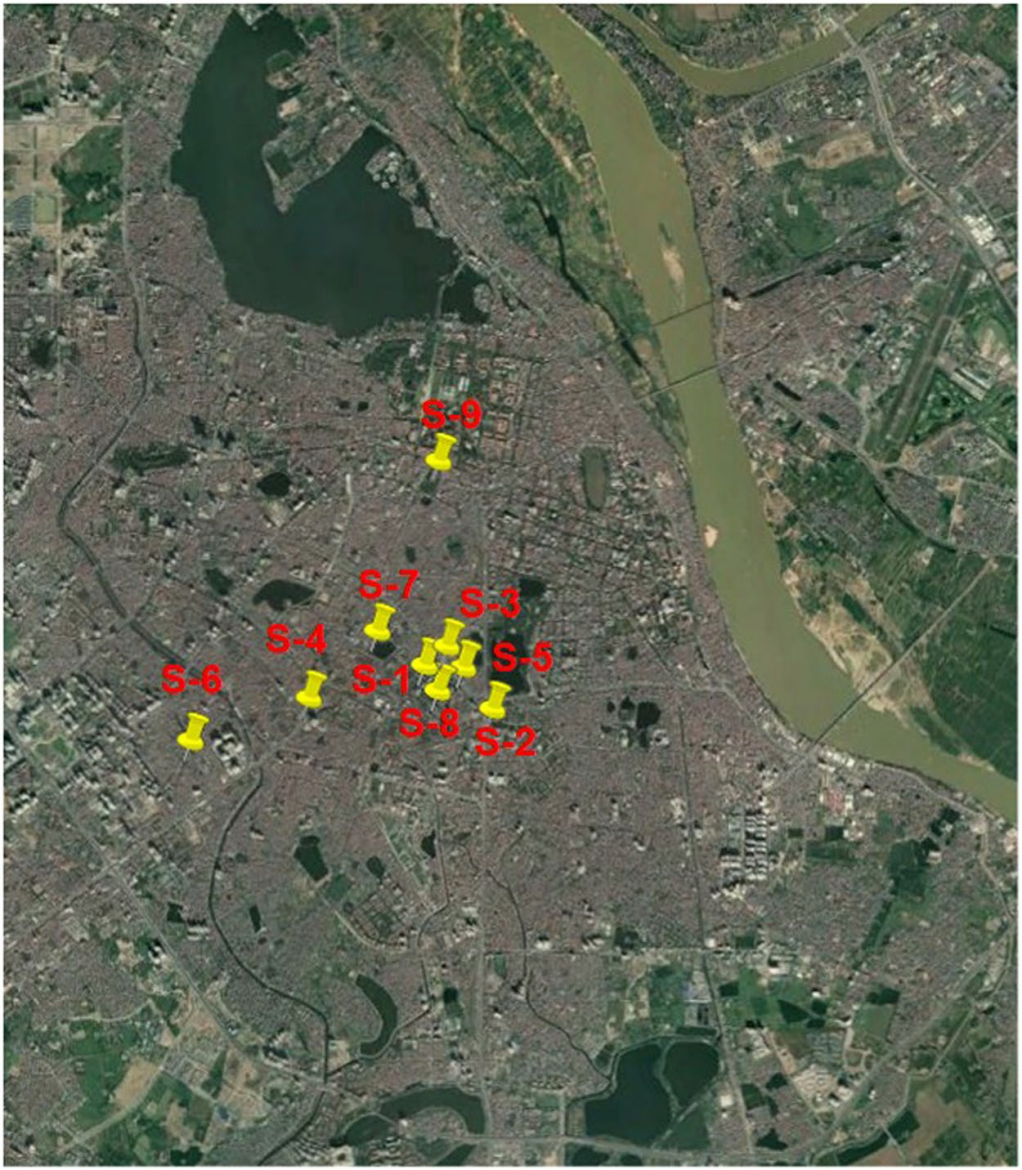
Referenced water depth from the flood event photos for 9 stations (background image map is obtained from the open source of Google Earth which data is from SIO, NOAA, U.S. Navy, NGA, GEBCO and image copyright belongs to 2012 Google, All the URL links of 9 stations are provided by Kha Dang-Dinh and Thanh Ngo-Duc). S-1: 0.47 m Website: First photography of https://vnexpress.net/tin-tuc/thoi-su/nguoi-ha-noi-cam-nhan-ve-ngap-lut-lich-su-2111640.html. S-2: 0.06 m Website: First photography of http://khoahoc.tv/ha-noi-doi-mat-voi-nguy-co-ngap-lut-38265. S-3: 0.1 m Website: It is named Xe buýt tắc nghẽn of https://thethaovanhoa.vn/xa-hoi/anh-doc-so-sanh-tran-mua-lon-o-ha-noi-voi-tran-lut-lich-su-nam-2008-n20160526192312704.htm. S-4: 0.7 m Website: Fourth photography of http://blog.tamtay.vn/entry/view/732375/Ha-Loi-2008-Co-ai-con-nho.html. S-5: 0.15 m Website: https://vi.wikipedia.org/wiki/T%E1%BA%ADp_tin:VCCI-DaoDuyAnh03112008.JPG. S-6: 0.35 m Website: It is named Đường Trần Duy Hưng of https://thethaovanhoa.vn/xa-hoi/anh-doc-so-sanh-tran-mua-lon-o-ha-noi-voi-tran-lut-lich-su-nam-2008-n20160526192312704.htm. S-7: 0.71 m Website: First photography of http://www.24h.com.vn/tin-tuc-trong-ngay/lay-dat-cong-vien-lam-bai-xe-dieu-cam-ky-c46a478826.html. S-8: 0.4 m Website: It is named Một đám cưới of http://vietnamnet.vn/vn/thoi-su/hinh-anh-kho-quen-khi-ha-noi-thanh-song-85178.html. S-9: 0.15 m Website: the time of 1:05 at https://www.youtube.com/watch?v=IJb4kQSOI54.

**Figure 4:**
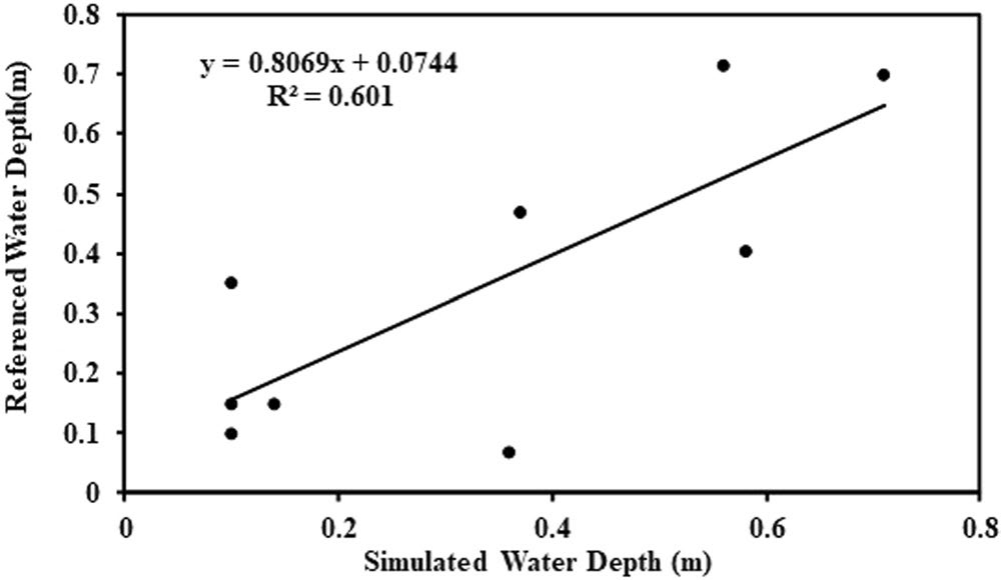
Regression relationship between simulated water depth and referenced water depth for 9 stations.

Flood inundation simulations were carried out for the central area of Hanoi. Four historical and extreme rainfall types are shown in Table 1. Historical rainfall for 2008 (R1) was used for calibrating the flood inundation simulation. Due to lack of digital observed water depth during flood events in 2008, water depth was estimated from the referenced photos by judging the water depth through referenced objects in the images. We use 9 referenced photos for calibration proposes in Fig. 3. All 9 points are in areas of high population density in the Hanoi central area. S1 is a roadside shopping area. The referenced water depth was judged as 2/3 of the bicycle tire with 0.47 m diameter. S2 uses referenced water depth of the height under the Asian man’s medial malleolus of 0.06 m. The water depth of S3 is judged from the pictures with 0.1 m which is about 2/3 road curb height. S4 uses the diameter of the bicycle tire of 0.7 m. The referenced water depth of S5 is the height of the curb of 0.15 m. S6 is the referenced water depth of about 1/2 car tire diameter of 0.35 m. S7 uses the water depth based on average upper thigh height of Asian women of 0.71 m. The referenced water depth of S8 is the average height of the tibia for Asian women of 0.4 m. S9 uses the referenced water depth of the height of the curb of 0.15 m. The simulated water depth was compared with the referenced water depth in Fig. 4. The simulated water depth in S1, S6 and S7 underestimated the referenced water depth. The simulated water depth in S2 and S8 overestimated the referenced water depth. Referenced water depth of S3 is the same as the simulated water depth. All other simulation results are close to the referenced water depth. The overall relationship between inundation simulation and referenced water depth yields an R^2^ of 0.6. This calibration result is limited to these 9 stations due to lack of observed water depth. The difference between referenced and simulated water depth is still large. Further studies on the modification of model parameters as well as the collection of observed water depth and urban sewerage system data are necessary to fully investigate the system dynamics.

**Table 1:** Rainfall type and inundation area in different depths.

Rainfall	Precipitation (mm)				Maximum Depth	Inundation Area (Percentage of 76.26 km^2^ (water surface included))			
	Total	1st day	2nd day	3rd day		0.3 m~0.5 m	0.5 m~1.0 m	1.0 m~1.5 m	>1.5 m
R 1	563.3	347	128.2	88.1	2.08	11.3%	9.1%	0.8%	0.1%
R 2	560.4	165.5	394.9	0	2.93	12.8% (13%)^*^	16.3% (80%)^*^	3.1% (268%)^*^	0.4% (209%)^*^
R 3	622	62	161	399	2.99	12.8% (13%)^*^	16.8% (85%)^*^	3.2% (284%)^*^	0.5% (216%)^*^
R 4	840	26.8	57.7	755.5	3.23	12.5% (10%)^*^	24.3% (168%)^*^	8.7% (939%)^*^	1.3% (820%)^*^

^*^Percentage numbers inside the bracket are meaning the increased percentage compared with the inundation area of R1.

Table 1 shows that the maximum water depth for R1 is 2.08 m, and the water depth for R2, R3 and R4 increased with change of precipitation intensity, amount and pattern. The total amount of R2 is similar with R1. The maximum water depth of R2 is almost 0.87 m higher than the maximum water depth in R1. The inundation area with water depth of 0.3–0.5 m is 11.3% of the total area with 76.26 km^2^ for the R1 rainfall. Inundation area increased to 12.8% for the R2 rainfall. The inundation area with water depth of 0.5–1.0 m in R1 is 9.1%, and it increases to 16.3% under R2. It increases slightly (0.5%) under R3 compared with R2. The percentage of inundation area with water depth from 1.0 to 1.5 m in R2 and R3 increased more than three times compared with that under R1. The inundation area for the water depth of 1.0–1.5 m in R4 reached 8.7%. The percentage of inundation area with water depth over 1.5 m was 1.3% under the R4 rainfall case.

**Figure 5:**
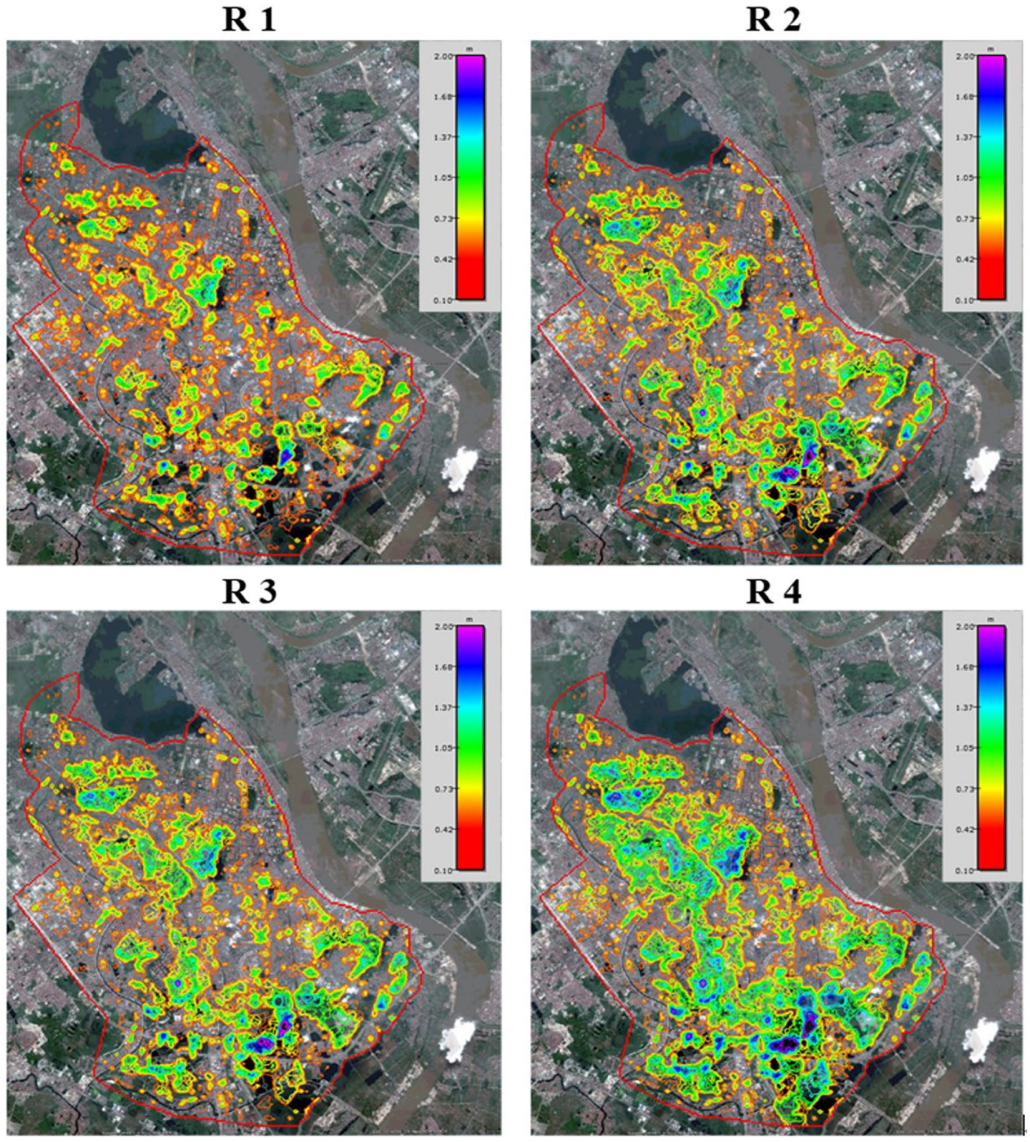
Maximum inundation depth and area under the four rainfall types of (**R1**–**R4**) (Source: Background image map is obtained from the open source of Google Earth which data is from SIO, NOAA, U.S. Navy, NGA, GEBCO and image copyright belongs to 2012 Google).

Figure 5 shows the spatial distribution of the inundation area. Satellite images were used for the background to clearly present the inundation results. The northwestern part of the Hanoi central area had the largest inundation area for the R1 rainfall case. The inundation area under R2 increased significantly compared with that under R1. The southeast part of the city exhibits the maximum water depth under the R2 rainfall case. The inundation area under R3 is quite similar with that under R2. The inundation area under R4 has almost double the inundation area than that under R3. The northwest and southeast part of the city experience the most inundation and the maximum water depth under the R4 rainfall case. Judging based on the 2010 land use map of the Hanoi central area, flood waters converge in the low elevation areas which saw shifts in land-use from water surface and agricultural to urban. Areas of dramatic inundation are in highly sensitive housing areas. The highest water depth was observed in public park and lake areas. Figure 5 and Table 1 show that flood inundation for R4 results in the most inundation area and highest water depth. This result provides analytical guidance suggesting that the Probable Maximum Precipitation can be used for predicting future urban flood inundation under extreme rainfall.

## Discussion

Spatial distribution of flood inundation under the four types of historical and extreme rainfall shows that the water depth and flood inundation area under two-day rainfall is higher than that under three-day rainfall with the same total rainfall amount. The level of rainfall intensity and pattern determines flood inundation risk. The annual three-day maximum precipitation with 200-year return period as well as the probable maximum precipitation are recommended to represent extreme rainfall for urban flood management. The rainfall intensity,duration and pattern is also important factors contributing to depth and extent of flood inundation, particularly as urban rainfall becomes more extreme under climate change^22,47^. Because urban flood frequency curves depend on rainfall amount and duration distribution^48^, urban flood prevention should consider the quantification of extreme rainfall events and return periods^49^. Some rainfall durations and intensities result in extreme early peak discharge in rivers^50,51^ which present a difficulty in pumping floodwater to nearby receiving bodies.

Due to a lack of detailed reservoir information, the flood inundation analysis in this study is only based on elevation. R2 shows the highest water depth in the southern reservoirs. Floodwaters are centralized in the reservoirs in R2 compared with in R1. For R4, water still flows into the central and southern reservoirs. Natural lakes or manmade reservoirs play an important role in reducing flood inundation risk in urban areas^52^ and adaptive reservoir management offers an opportunity for flood mitigation under climate change^53^. The capacity and numbers of reservoirs have a direct effect on reducing flood inundation risk. Improved reservoir management can help reduce flood inundation in urban areas^54^. Multiple reservoirs and operating rule curves are vitally important for water supply balances and the protection of downstream ecological systems and flood control^55^. Integrated reservoir operation should be combined with real-time monitoring systems to protect against flood events and multi-purpose reservoir systems can be constructed or adapted for flood control. Although hard infrastructure around reservoirs and dykes can defend against urban floods, increasing or reestablishing connectivity of reservoirs and rivers can mitigate flooding and overbanking.

Low elevation and high population areas are uniquely vulnerable to urban floods and economic destruction. The flood inundation simulation of 2008 shows that most of the inundation areas are in high population density areas and caused major economic loss. Results show that the most inundated areas are in the low-elevation housing area for the R4 case. Some human activities that increase flood risk are pumping underground water and building large structures leading to land subsidence and reduced elevations. Lack of observed river channel cross-section data and observed discharge made it difficult to calibrate river discharge in this simulation. Further research can be done to calibrate flood inundation models using observed discharge data. Due to limited observed data, the Flo-2D model for the Hanoi flood inundation did not consider real conditions such as height of buildings and boundary conditions. The results obtained from Flo-2D have numerous uncertainties related to the model structure, data input and boundary conditions. Exploring and resolving these uncertainties point to an important area of further study.

Present flood management plans make reducing flood inundation difficult in the context of the four types of extreme rainfall. Results suggest that setting up pump stations to deliver water to the Red River, reservoirs or underground water storage facilities would be effective. This study investigated installation of a pump station to discharge floodwater to the Red River. The pump station was not effective to reduce the inundation area and water depth due to the low elevation of the urban central area. Underground water storage offers a promising approach to reduce inundation area and water depth in high population areas, but with a high cost. Rainfall harvesting systems could store some of the water and delay the water inflow to the urban sewerage systems. Large-scale shifts in land use are essentially necessary for defending against urban floods^56^. Land-use management in urban areas can help store rainfall (e.g. in reservoirs and wetlands) and manage infiltration to the underground soil by green land. Reducing construction in natural floodplains can enhance natural flood attenuation capacity of rivers in urban areas. Prone areas of low elevation can be converted into multi-purpose reservoirs with integrated flood control operations. Wetland restoration is another option for reducing flood inundation, improving ecosystem health and regulating urban hydrologic extremes. Risk analysis based on new data, theoretical development and boundary conditions can help in evaluation of existing flood protection systems for long term management decisions^57^. The increase in expected annual economic losses under climatic change should be considered in future adaptation for flood prevention measures^12^. Adaptation strategies like pipe enlargement and local infiltration can be used to integrate open drainage basins in urban recreational areas^58^. Sustainable flood management should include the present, near future (50 years), and future (100 years) period. Present flood management can incorporate modern online monitoring systems to predict flood inundation at the catchment scale. Such systems should include an online rainfall monitoring system with high accuracy and short time steps (10 minutes or 1 hour) for the entire catchment, an online water-level monitoring system for the upper stream and an online water depth monitoring system for urban areas. Near-future flood management should provide flood risk analysis at the catchment scale and flood inundation simulation of urban areas based on extreme rainfall for more than 100 years return period. Green roofs and vertical greening, rainfall harvesting systems and underground water storage offer opportunities to protect prone areas. For the future, reforestation in urban areas and upper stream reaches as well as sustainable dam construction in appropriate locations can contribute to sustainable flood management.

This study presents a flood inundation model with acceptable calibration results compared with referenced water depth. Four types of historical and extreme rainfall yield different depths and area of flood inundation in the Hanoi center area. Higher intensity extreme rainfall can cause higher water depths and a wide inundation area. Reservoirs play an important role in reducing flood inundation risk in urban areas. Pump systems, green roofs and vertical greening systems, rainfall harvesting systems, and underground water storage systems offer the potential to reduce water depth and inundation area. Sustainable urban flood management should be integrated into catchment management, flood management infrastructure construction, online monitoring systems, flood inundation modelling, early warning systems and urban development plans. This study could be improved by taking more observed water depth data to calibrate the flood inundation model. Climate and environmental change should be considered in future studies. Further research should investigate the effect of intensity and patten of extreme rainfall on inundation depth and area.

## Methodology

### FLO-2D Model

Flo-2D model software, developed by O’Brien *et al*. (1993), can be used for the simulation of floods and debris flows. This model has been applied widely for flood risk management, urban floods, mudflows, and debris flows. Flo-2D comes in two version, the Flo-2D Basic Model and Flo-2D Pro Model. The Flo-2D Pro Model is a professional model with more sophisticated attributes, including storm drain modeling with surface-water interface, dam and levee breach, groundwater exchange with surface water, sediment transport, mud and debris flows. In this study, Flo-2D Pro was used for simulating flood inundation under four extreme rainfall types. The two-dimensional governing equations are the continuity Equation (1) and dynamic wave momentum Equations (2) and (3):

1 $$\frac{\partial d}{\partial t}+\frac{\partial d{W}_{x}}{\partial x}+\frac{\partial d{W}_{y}}{\partial y}=e$$

2 $${L}_{fx}={L}_{Ox}-\frac{\partial d}{\partial x}-\frac{{W}_{x}}{g}\frac{\partial {W}_{x}}{\partial x}-\frac{{W}_{y}}{g}\frac{\partial {W}_{x}}{\partial y}-\frac{1}{g}\frac{\partial {W}_{x}}{\partial t}$$

3 $${L}_{fy}={L}_{Oy}-\frac{\partial d}{\partial y}-\frac{{W}_{y}}{g}\frac{\partial {W}_{y}}{\partial y}-\frac{{W}_{x}}{g}\frac{\partial {W}_{y}}{\partial x}-\frac{1}{g}\frac{\partial {W}_{y}}{\partial t}$$

where *d* is the flow depth, *t* is time, *W*_*x*_ and *W*_*y*_ are depth-averaged velocity components along the x and y coordinates, *g* is gravitational acceleration, *e* is the excess rainfall intensity which may be nonzero on the flow surface^59^. The friction slope components *L*_*fx*_ and *L*_*fy*_, bed slope *L*_*ox*_ and *L*_*oy*_, pressure gradient, convective and local acceleration terms are presented in Equations (2) and (3).

### Historical and Designed Future Extreme Rainfall

**Figure 6:**
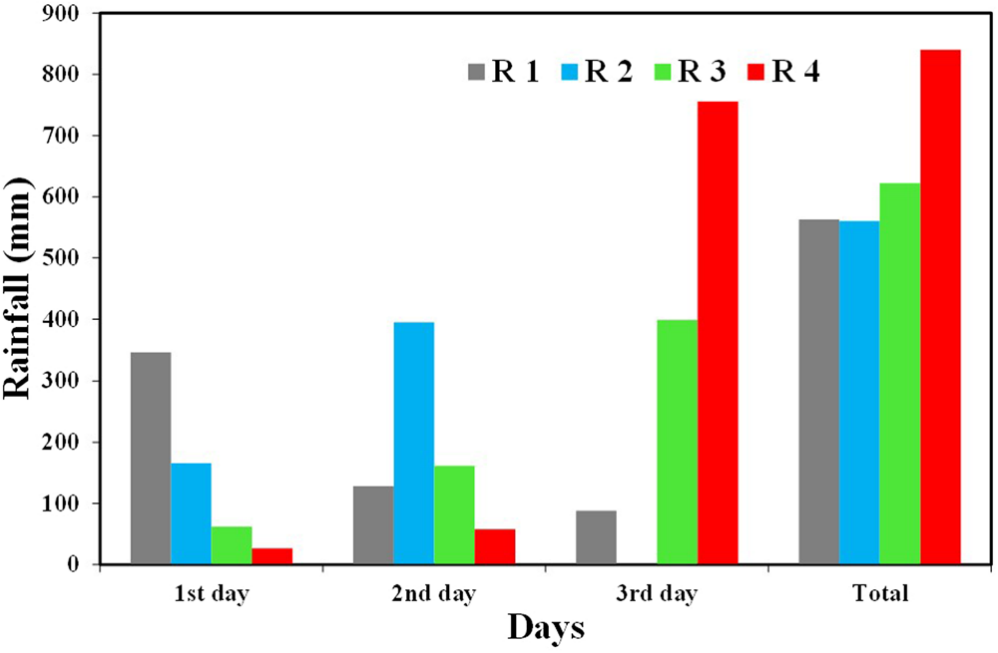
Four types rainfall for the flood inundation assessment.

This study uses four historical and extreme rainfall events for rainfall input. Four historical and extreme rainfall events are designated R1, R2, R3 and R4 in Fig. 3. R1 is the actual precipitation of the 2008 Vietnam Flood (October 31, 2008 to November 2, 2008) used for model calibration. R2 is the actual Hanoi precipitation of the historical recorded two-day maximum precipitation for the period of November 9 to 10, 1984 for historical reference (1961–2008). R3 and R4 are designed to three-day duration precipitation events to estimate the most extreme flood inundation conditions. R3 used the Frechet 3P probability distribution function to calculate the probable precipitation within return period of 200 years. Observed daily precipitation data (1961–2008) from the LANG station in Hanoi was used to analyze the probable precipitation R3 using the Standard least square criterion (SLSC) goodness of fit test. Annual maximum three-day precipitation with 200-year return period was selected for extreme rainfall conditions of R3. Extreme rainfall event R4 used the probable maximum precipitation method to get the physical probable boundary of precipitation for Hanoi. Figure 6 presents all four rainfall scenarios. Precipitation for one day is assumed to have fixed intensity per hour (one-day precipitation/24 hours).

### Frame work of flood inundation assessment

**Figure 7:**
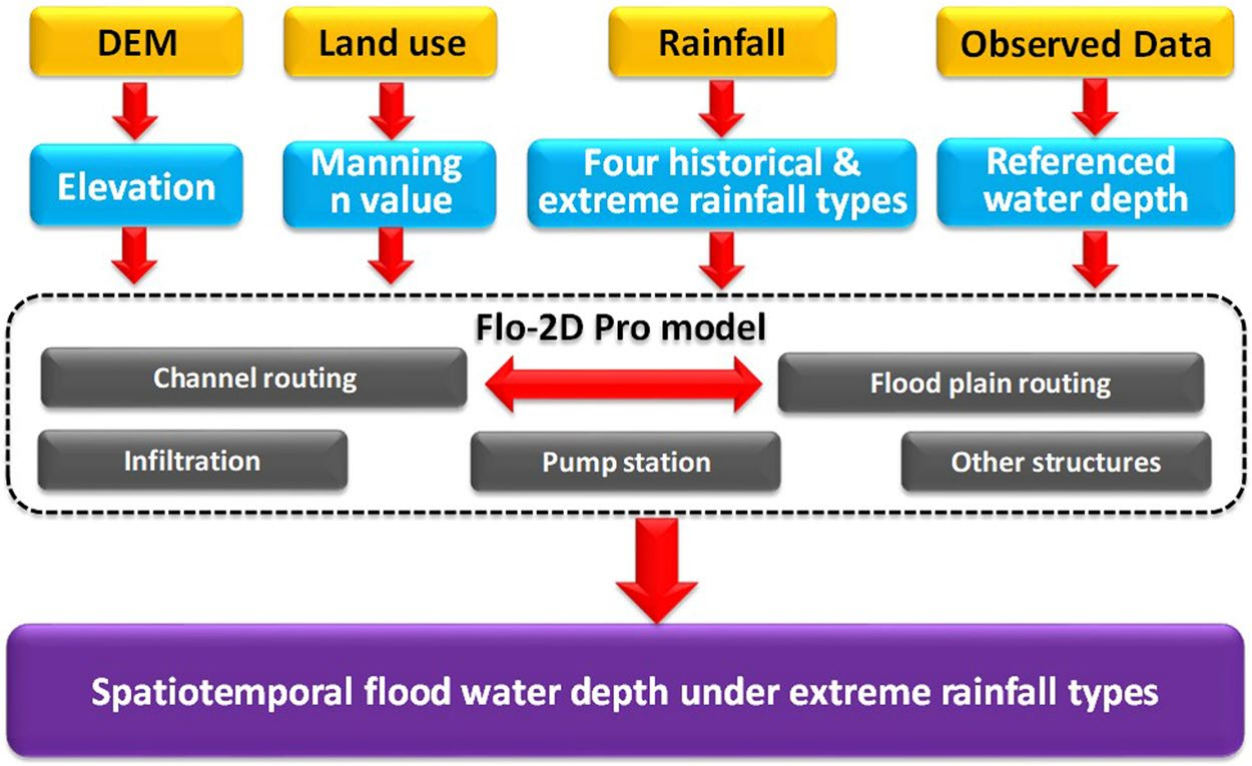
Frame work of flood inundation assessment under the historical and extreme rainfall in Hanoi central area.

Figure 7 shows the framework for flood inundation assessment under four historical and extreme rainfall events in this study. The framework of this study is divided into three parts: data preparation, model application, and result comparison.

1. Data preparation. High resolution Digital Elevation Model (DEM), land use, rainfall, and other observed data was collected for this study. The high-resolution DEM data was converted into ArcInfo ASCII grid elevation data. The land use map is assigned Manning n values and infiltration-related parameters based on previous studies and the Flo-2D Pro manual^60^. We selected four historical and extreme rainfall events which were explained in Section 4.2. Observed data for model calibration was based on referenced photos taken during the 2008 flood events. We judged the referenced water depth for comparison to the simulated water depth.
2. Model application. Elevation input data and hydrological parameters were assigned for each grid cell. The elevation of grid cells for the river channels was modified from upstream to downstream. Infiltration of each grid cell is based on the Green Ampt method^61^. The flood inundation model was rerun using the different rainfall scenarios. The pump station location and capacity were input to the model.
3. Result comparison. Simulated water depth of 2008 historical flood event was compared with the referenced photos for calibration. The fixed flood inundation model was used to analyze water depth and inundation area under 3 extreme rainfall types. The simulation results under four historical and extreme rainfall were compared in this study.
